# Observations on Convulsion, as a Consequence of Disease; Illustrated by Cases

**Published:** 1810-06

**Authors:** J. Howship

**Affiliations:** Surgeon


### Observations on Convulsion, as a Consequence of Disease; illustrated by Cases.

By Mr. J. Howship, Surgeon.

In Continuation.

### *A Case, in which very slight Derangement in the Structure of the Brain produced Convulsions, eventually terminating fatally.*

CAPTAIN F. T. of the 82d regiment of Foot, a gentleman twenty-eight years of age, had been long troubled with an hæmorrhoidal complaint; to this he had been subject for some years, but it is here mentioned merely on account of its having been latterly attended with occasional losses of blood. In this point of view, it related to complaints much more important.

The hæmorrhoidal disease had produced several tumors around the verge of the anus, of considerable size when distended with blood. These were rarely attended with any inconvenience beyond that of the patient being obliged to lay down for a quarter of an hour or more, after having passed a stool. In the horizontal posture they were very readily returned, and gave no more trouble till the next day.

On Friday morning, October 7, 1808, while the battalion lay at Scarborough in Yorkshire, an unusual flow of blood took place on the water closet; he supposed that he might have lost as much as half a pint of blood by this accident. He did not, however, experience any sense of faintness, nor the least inconvenience, from the hæmorrhage, but, on the other hand, when he left the mess in the afternoon, he dressed for the assembly, and during the evening danced for nearly two hours.

About one o'clock he left the company, and returned home to his apartments, sat down, and was listening to a young gentleman who was reading to him, when in a moment his features became frightfully changed and distorted, and his breathing attended with a most unnatural sound. At the first glance, his brother officer concluded that he must be joking, and began again to read, when in a few seconds he fell into a state of universal spasm and convulsion, and was in an instant thrown down from his chair. The attack was rather that of rigid spasm than of violent and convulsive action.

In a few minutes he came to himself, and appeared to be quite recovered; he felt no pain, and was not in the least degree aware of what had happened. Before he could could be assisted up stairs to his chamber, and got into bed, he was again attacked, and remained as before for some minutes quite insensible, and in a state of partial convulsion; after which he again revived, and recovered his senses. He had not been in bed above half an hour when he complained of sickness, and soon after threw off the contents of the stomach; but this afforded no evidence of any morbid condition of the bilious fluid.

The fits still continued to return about every half hour. A drachm of the ammonia prep. was given in a draught, but this apparently did nothing. Aromatics also were advised, and swallowed, but without any benefit. At day-break, he said he felt himself becoming easier, and more composed. Soon afterwards he slept without any disturbance for several hours.

Saturday, October 8. He passed the day perfectly free from convulsion, said that he was better, and that he found his head clearer than it had been during the night; but that he felt himself very weak. This day, at the desire of the first medical gentlemen of the place, he took the saline draught, with a pill of calomel and antimonial powder, of each one grain. These medicines were repeated every three hours. It was expected, that in all probability they would act upon the bowels in the course of the afternoon; but this did not happen, and he had no stool on this day.

Several times towards evening he complained of great pain in the head, and the countenance became considerably changed, appearing overcast, dark, and suffused. About eleven at night the fits returned as before, but with rather more violence.

Sunday, Oct. 9. The attacks of convulsion continued to return, frequent and severe, till the hour of four this morning, when the disturbance seemed again to subside. Soon afterward he fell asleep, and some hours of tranquil repose enabled him to pass the day, to all appearance, in an improving state. He considered himself to be better than he had yet been. At the commencement of the disorder, the pulse had been harder and rather fuller than natural, but had never exceeded 96. The salines had apparently been the means of rendering it softer and smaller than it had been before. Towards the afternoon of this day the appearance of the face altered very much, the complexion assumed a darkened colour, almost livid, so as to render evident that the head was now overloaded with blood. Without waiting a consultation, a very large blister blister was immediately laid between the shoulders, and a cathartic mixture given. Towards evening he had a stool. At nine this evening twelve ounces of blood were taken from the arm; and this, he said, "very much relieved his head." Soon after he became somewhat faint; and while this state continued, he several times experienced slight tremors, but no decided return of convulsion. In an hour these appearances vanished, the cold perspirations were not renewed, and from midnight he slept very well.

Monday, Oct. 10. Much better; had rested very comfortably, and was up almost the whole of this day. His only complaint was of weakness and soreness in his flesh. During the forenoon, the tartarized infusion of senna was repeatedly taken till it operated about three in the afternoon. He experienced no return of his complaint. In the evening he retired, and passed the night in comfortable repose.

Tuesday, Oct. 11. Extremely well; had passed a good night, was up early, and made a very sufficient breakfast. During the forenoon he had a motion. There was at this time not the least degree of heat upon the skin, nor any mark of feverish disposition. For dinner he ate a small piece of rump steak, and drank (what had been proposed, but most expressly forbidden) a glass of porter. He retired to his chamber about ten in the evening, just before which he had another natural motion.

About eleven o'clock he was heard to get out of bed, and move about the room; but he made no particular noise, nor any complaint. However, as the people below were very kind and attentive to him, they took it for granted that he was again ill, and therefore hastened up stairs. The door of his chamber he had locked, but they succeeded in getting into the room by another way, and found him standing opposite the door, endeavouring in vain to get the key into the lock, in order that he might open the door. He answered every question with tolerable precision, but it was evident enough that his intellect was confused. The bed-clothes were found about the room, and the whole bed so much disordered, that in all probability he must have had a fit before any one was aware of his relapse. As soon as possible he was again laid in bed, where he immediately fell into another fit, in which the convulsion seemed to spend most of its force upon the left side of the body. It came on just as he had laid apparently disposed to rest and sleep. The first token of the approach of the paroxysm was in the eye-lids, which were slowly raised raised up, and opened wide, discovering the eyes turned by a convulsive action, first upward, and then toward the left side, where they remained fixed while the fit continued; the mouth also was violently contracted, but drawn aside to the left ear, the nose and skin being distorted in a similar manner.

By the time these appearances had taken place, the face was slowly moved, with an irresistible force, round to the left side; and now the arms were thrown violently back, and the body and extremities shaken alternately by a sort of spasm, with a general tremulous agitation of the whole frame. The respiration was laborious and quick, the air rushing with an audible sound through the nostrils. In these fits, as in epilepsy, the tongue was sometimes bitten, so that the saliva occasionally passing the lips was sometimes tinged with blood. The action of the heart in the paroxysms just described, was manifestly disturbed, altho' the change seemed to lay in the increased hardness rather than in the quickness or irregularity of the pulsations.

The fits continued to return during the night, with the interval of twenty minutes only; they ceased about four in the morning, after which time he slept without uneasiness or disturbance for several hours.

Wednesday, Oct. 12. About nine o'clock this morning he awoke, much refreshed by the long sleep he had enjoyed, but complained of great pain about the left shoulder and arm, down to the elbow. He said, however, that he found a proportionate degree of ease in his head, and observed, that he was very glad to find that the complaint had left his head, and passed down into his arm. Upon examination, the parts about the deltoide muscle were found very tender and painful to the touch; added to which, he found that he had lost all power of moving the arm; and when desired to raise the left arm to his head, he was obliged to lift it with his other hand. On comparison, it appeared that the affected arm was much paler as well as colder than the other.

In the fits that took place at this period of the complaint, the left arm, from being as at first, most subjected to the force of the spasm, was now almost motionless, the only perceptible action being confined to the muscles bending the fingers. The leg on the left side had undergone exactly the same changes, but in a less material degree.

Thursday, Oct. 13. The fits came on this morning very soon after he awoke, and continued to return at their former intervals. This morning, for the first time, he had an involuninvoluntary passage of the urine. It had been proposed that Dr. Simpson, a physician of very high character in the neighbourhood of New Molton, should be sent for, and about eleven he arrived. With great judgment, he considered that the disease was a modification of epilepsy, and that it must be treated on the same principle, being in all probability connected with effusion, either of blood or serum, upon some part of the brain, especially as the spasm had more particularly affected one side of the body. In consultation, it was determined that the only system here was that of depletion; and from the unusually small size of the external jugular vein, the opening the temporal artery was recommended. The patient, just recovered from a fit, was assisted out of bed into an easy chair; and as it generally has been found, that where paralysis has taken place on the one side of the body, it has been consequent to some derangement upon the opposite side of the brain, the right temporal artery was preferred, and opened.

In the operation, the blood sprung forth very abundantly, and during the time that the artery was bleeding he had no less than four fits in succession, which, notwithstanding the abstraction of a part of the circulating blood, still retained exactly their former appearance and character; although he several times said in the intervals, that the bleeding had done him good, and that his head was very much relieved. The pulse rose perceptibly, and became more free and full, while the pulsation had more of a natural softness and action of the vessel than before. More than twenty ounces of blood were allowed to flow off in a free stream, without the least appearance of faintness; pressure by a compress and bandage of broad tape was then so applied as to prevent any further effusion.

After finishing this operation, the convulsive attacks remained much the same as before in point of violence, and still returned every twenty minutes. He was carefully laid in bed again, and a strong drastic enema thrown up, which soon after operated.

At three this afternoon the fits subsided, and he fell into a dose; when, some time afterward, he was disturbed, his ideas were perceptibly less collected than heretofore, for when he spoke, he was unable to express himself coherently. About this period it was very curious and highly interesting to observe the various degrees of excitement that existed in various parts of the brain; or, to speak with more certainty, in the different organs of sense.—

From

From several circumstances, it was clear that the sense of hearing was infinitely more acute than it ever was known to be in its natural state. The sight, on the contrary, was just as much impaired as the hearing was improved. He was not at this time able distinctly to make out his friends, who came to his bed-side to see him, although he always told the name the instant he heard the voice. The eye had been at one time so sensible to light, that a candle even in the neighbourhood produced uneasiness; but now, the brightest candle might be brought close to his eyes, without his seeming to perceive it. It is remarkable, that notwithstanding this loss of vision, the pupil of the eye still was observed to contract and dilate, in exact proportion to the intensity of the stimulus by which it was thrown into action.

This evening the spaces between the bandages that secured the temporal artery were shaved, and a very large cap blister was applied over the whole extent of the scalp; at ten o'clock the fits again returned with intervals of a quarter of an hour, and they soon afterwards followed each other at periods still shorter; he complained of great and distressing thirst, and his constant anxiety and desire was for "more whey."

A cathartic mixture with infusion of senna and Epsom salts was given every hour, but did not operate; several times this evening he felt a shooting darting pain in the divided artery, which he said was just as if he was being cut again with the lancet.

For half an hour this evening he was making unavailing efforts to pass his water; after this time it came away, and without pain he evacuated more than a pint; since the closing of the artery, the pulse had remained much softer than before the operation, but still it was quickened. At all times, when questioned, he said that his head was much relieved, by the last bleeding particularly.

Friday, Oct. 14. The fits all the early part of this morning had returned with very short intervals, and in the paroxysms the right arm and leg only were affected by the spasms, the opposite members having totally lost their power of action; the head and face continued to be as frightfully distorted as ever.

About half past three this morning he lay back in the bed, and stretched himself out; after the fit was over he heaved a deep sigh, and then lay for some time at perfect rest; breathing did not perceptibly recommence for several minutes, he changed colour, and it was supposed that he was expiring.

expiring. In a few minutes, however, the breast again heaved, and the fits continued at the rate constantly held for the last forty-eight hours.

With this temporary state of exhaustion, the feverish thirst subsided; his power of expressing his feelings was as perfect as before, but he had no desire to drink, nor would he swallow any thing when offered; at mid-day the thirst began to return upon him again, and before the evening, had reached its former degree of intenseness.

At eight o'clock this evening the pulse was weak, 120. The scalp above the bandage had become tumid, and of a much darker colour than the other parts of the skin; there was a copious and offensive discharge from the blistered surface. Several times to day he felt a desire, without having the power, to expel his water; during the evening it repeatedly flowed off involuntarily, and twice without his seeming conscious of it.

The tumour and puffy state of the scalp from the tightness of the bandage was this evening considered to require some attention. It was certainly too early to remove altogether the pressure upon the artery, while it was nevertheless necessary that so strict a ligature should no longer be allowed to remain. Fortunately, however, it appeared that even if the binders were carefully divided just over the forehead, there might still be sufficient support to the compress, from the adherent state of the tapes around to the integuments of the head. This therefore was done, the divided edges of the tapes flew asunder, but in no other point were the applications disturbed. In less than an hour the best effects resulted from the removal of the ligature, while yet all remained safe in relation to the artery. He felt much relief from the removal of the stricture about his forehead.

In the course of this evening and night, the attacks of convulsion were more frequent than ever, but of shorter duration and less intensity, attended with more relaxation of skin, less congestion in the extreme vessels, as marked by the livid colour of the face and lips, and less injury to the intellect than formerly. The paroxysm did not continue above a minute on the onset of the fit; the skin now fell suddenly white and pale, and a copious sweat broke forth. During the progress of this night, the thirst was very distressing; the forehead and face were frequently incommoded by transient flushes of heat; there was still complaint of pain in the ear, by the divided artery; he frequently said, "my thirst is insatiable." The early early re-establishment and controul the mind exerted over the body was truly astonishing about this time, for even while the convulsions were yet upon his limbs, and his respiration stertorous, and to a high degree laborious, he would reach out his trembling hand for the handkerchief to wipe away the moisture from his lips.

He frequently desired to be sat upon the close-stool, but at those times never was able to pass any thing, nor had all the laxative draughts taken within the last two days produced any motion, although he had swallowed more than three ounces of purging salts, independent of other drugs. The periods of interval between the fits through this night were fifteen, ten, eight, and once only five minutes. The extremities were pale, moist, and cool, a free perspiration breaking out after every paroxysm. During the fit the pulse was perfectly regular, but very tense and hard, from the spreading influence of that spasmodic disposition, which seemed now to pervade nearly every part of the body.

Saturday, Oct. 15. This morning the compress was removed, and the remaining parts of the bandage taken away from the temple. The wound was found in a fair state of suppuration; the day passed rather promisingly; the pulse beat 100 in the minute. This morning he had a stool; his mind was extremely tranquil, and more collected than it had been for some days past; the longest interval this day was near six hours.

This evening he fell into a very unusually sound sleep, snoring sometimes very loud. From this the servants imagined that he was much better; but his state appeared to be more a comatose insensibility than a natural sleep. At four this afternoon his pulse was 92 only. It was seven o'clock when the lethargic sleep alternated with the fits, which sleep gradually wore off again.

In consultation this day, it was proposed that a quantity of mercury should be introduced into the constitution, on the ground of its possibly exciting a salutary activity in the absorbent system. The plan had, on a former day, been suggested; and now, as it met with the approbation of all, it was acted upon. Five drachms of the mercurial ointment were immediately rubbed in, and before midnight four more were consumed, so that nine drachms were rubbed in about the thighs this first day of its exhibition.

Between seven and eight this evening it was certainly desirable that more blood should have been taken away, on account of the evident state of comatose sleep by which he he was overpowered. This desire however was over-ruled; for when the consultation, an hour afterwards, was held, his apparent state had become so much altered, that it did not meet the opinion of the majority. He was now quite watchful and reasonable, although only an hour before, shouting aloud in his ear, and even shaking, would not rouse him. At nine he relapsed into this apoplectic state, with a stertorous respiration, and an even pulse at 100. This state soon afterwards again subsided, the fits returning precisely as before.

Sunday, Oct. 16. This morning a draught, containing scammony, gamboge, and calomel, of each five grains, was given; this produced a sharp action in the bowels; several involuntary stools were passed during the day. First came offensive feculent matter, afterwards the limpid fluids secreted into the intestines. By the operation of so violent a medicine, it was natural to expect what happened, a very material reduction in the strength of the pulse, still he said he felt much better from the physic; the fits continued their old course, returning at very short intervals. On this day one ounce more of the mercurial ointment was rubbed in upon various parts of the body. The pulse now varied from 100 to 120; the bowels greatly relaxed, and very active. He now complained of no pain or uneasiness in the head, but felt constant pain in the upper part of the paralyzed arm, of which he could not move a finger; the foot and leg on the same side were becoming œdematous.

The attacks of convulsion were now somewhat altered; the eyes and face were still, as formerly, affected first; but the chest was now fixed, and the breast did not heave till the close of the fit. This alteration had taken place by very slow degrees; the respiration formerly had been continued, but with labour and foaming at the mouth, and some degree of stertor. Now, on the contrary, by an uniform and obstinate spasm which seized upon the whole of the muscular parietes of the chest, the air was expelled from the lungs with a sort of scream, and the thorax was apparently compressed to the utmost degree. During this state of spasm, the face and lips assumed a dark and even livid colour; but as soon as the violence of the paroxysm began to abate, which generally took place in about half a minute, the breast again moved, and the air, by short starts, was again drawn into the lungs, by a sort of fluttering action of the muscles. This motion of the muscles of respiration, after a few minutes, settled into a very languid guid state of their proper action. The vital organs generally decline together; the pulse, for the space of some few beats, could not now be felt at the same instant when the spasm was at its height. He observed to day, that he did not think he should recover.

Monday, Oct. 17. He remained much the same. The blister upon the head was removed, and a large plaster of the savine ointment substituted. Where the bandage that secured the artery had passed under the chin, there was now a sore and inflamed surface, from the flowing of the discharge from the blister. From the same cause, the conjunctiva membrane of the right eye was now very sore, painful, and inflamed. The mercurial frictions were still continued, at the rate of two drachms every two hours.

Tuesday, Oct. 18. Ever since the last purging draught was given, the bowels had been in a very disturbed condition, and still the discharges were fluid, frequent, and involuntary. From the quantity of watery fluid voided in the alvine discharges, it would appear that the fluids must have been poured into the intestines from the want of that due tone in the exhalent vessels, by which that canal, in ordinary cases, is enabled to return to its accustomed state of action, after the operation of a purgative medicine; the discharges were flatulent and fluid, without any fæcal matter, but still very copious.

He appeared now to be scarcely conscious of the manner in which his motions passed; in fact, both the alvine and urinary fluids now flowed off without his making any sign. The pulse varied from 100 to 120. He complained of very great pains in his bowels and in his head; towards the evening the pains in the abdomen were much diminished, but still he suffered from sensations of uneasiness about the forehead and over the eyes.

He had been persuaded to eat a very little piece of a light bread pudding, but it was only in the way; he felt and complained of it as a load upon his stomach the whole of the afternoon. The wound in the temple was now dried up, appearing just as if the discharge had dried on, and all further action was suspended in the part. The mercurial frictions were still continued.

Wednesday, Oct. 19. During the night, the fits had continued every ten minutes; the contents of the bowels frequently were passed involuntarily; in the evening and night closing this day, the symptoms of extreme debility in the constitution were so greatly increased, that the pulse, which in the continuance of the spasm was much oppressed, and did not beat more than 80, or even 70, in the minute, just after the return of the breathing, hurried on at the rate of 160; although before the accession of the next following fit, it had generally subsided down from 160 to 120 in the minute. The face also was now every hour assuming an aspect more and more livid in the moments of convulsion. In the fits there was at this time scarcely any perceptible vibrations in the limbs, although the body remained still as much under the influence of spasm as ever, so far was the nervous energy and muscular force exhausted. In the course of this day ten drachms more of ointment were rubbed in upon the back and thighs, making the quantity actually absorbed within five days, equal to at least several ounces of strong mercurial ointment, which never produced the least degree of excitement that could be distinctly ascertained, either in its effects upon the mouth, or even in giving firmness to the pulse. This last circumstance was noticed as curious by all who saw him.

Thursday, Oct. 20. In the night previous to this day, the fits continued, succeeding each other latterly with an interval of about twenty minutes. At six he had a fit, and after it desired some whey; about twenty minutes after he had another, and again rested. Ten minutes before seven he turned his head, and his eyes were observed to open in silence as before, and at the same moment respiration ceased. Instead of the spasm that till now had attended at this period of the attack, not any tremor could be perceived, the countenance fell, the colour left the cheeks, and death terminated his sufferings.

It was remarkable that the moment the face assumed its livid aspect, at the time of his dissolution, a sudden gush of blood ran down the side of the head, as if from the cavity of the ear; this hæmorrhage was to a considerable extent.

### Examination of the Body.

On opening the head, the first remarkable appearance was an adhesion between the dura mater, pia mater, and brain. This adhesion was situated upon the fore part of the right anterior lobe of the cerebrum; it was in extent equal to a crown piece, and opposite to the anterior extremity of the falciform process. This adhesion was principally on the right side; but on a careful examination it was found to a very small extent to exist on the left side of the falciform process also.

The connection between the brain and its membranes was at this part so very firm, that in raising the dura mater from from the brain, a portion of the cortical substance was torn up with it; this circumstance at first suggested a doubt, whether a small absces might not have existed here.

The inflammation, however, producing this appearance, was not presumed to have been of any recent date, for, notwithstanding the firmness of the union was very remarkable, yet no increase in the number of red vessels was apparent, nor any altered tinge in the general colouring of the parts, to indicate a late increase in the action of the vascular system of this organ.

Considering the general state of the blood vessels spread upon the surface of the brain in the pia mater, it might be imagined that the cerebrum was overloaded to a certain degree, although no trace of extravasation could any where be detected.

On the internal surface of the frontal bone, near the anterior termination of the falx, was found a small adherent spicula of bone. It lay in a line with the sinus, and close upon its margin. It was rather less than half an inch in length, and its elevation beyond the inner table of the cranium one-tenth of an inch; it is therefore presumed, that this process could not have conveyed any irritation or prejudice to the welfare either of the brain or its membranes. There were, however, different opinions upon this point.

Whether this spicula of bone was a fragment broken off from the inner table by external violence, or whether it might have a modification of the ossific process from diseased action of bone, seemed to be a question involved in much uncertainty. The general idea was, that of its having at some time been a detached portion of bone, afterwards becoming again united to the skull. But an objection to such supposition was the exterior surface, and indeed the surrounding parts of the same bone, bearing no visible mark of depression, fracture, or even fissure. Thus much, however, is certain, the spicula was situated immediately exterior to those adhesions that connected the membranes to the brain, while from its not possessing the least asperity at either end or on its edge, it could not be supposed that the adhesions were owing to any irritation from the presence of this minute fragment of bone.

The plexus choroides in each lateral ventricle contained a small, softish, vascular tumour about the size of a hemp seed, having the feel of a scrophulous enlarged gland. The ventricles were by no means loaded with fluid; on the contrary, they contained a less proportion than is very generally found.

The pineal gland contained an earthy or calcarious matter.

All the remaining parts of the brain were attentively examined, without any further derangement in its organization being discovered.

The contents of the thorax were sound.

In the abdomen the only appearance of disease that was observed, was an increase in the vascularity of the peritoneum lining the pelvis. The hæmorrhoidal vessels spread upon the rectum were not more than commonly numerous, nor in the least disposed, as they had been externally, to the production of varicose tumours.

The temporal artery, low down, being opened, and a probe gently passed up it, made its appearance without feeling the least impediment, out at the divided orifice of the tube; the artery was now laid open, and it was ascertained that there was not the least trace of a coagulum, therefore, without a doubt, the hæmorrhage that took place at the moment when the congestion in the vessels of the head was extreme, arose from the slightly adherent sides of the previously compressed artery giving way.

From the above examination it appeared, that the principal, perhaps the only material injury in the state of the brain, consisted in the adhesion opposite the frontal bone. This might have been the result of inflammation, consequent to a blow, particularly as there was another appearance, almost proving that the bone had, at some former time, sustained a shock. The probability however of this mischief having been the result of an accidental violence, was still further strengthened by several men in the battalion recollecting, that about seven years before, while the regiment was in Ireland, he had been thrown from his horse, and received so violent a blow upon his forehead, that he for some time lay senseless, and was confined for three weeks afterwards.

It is highly curious, somewhat unaccountable, and certainly very much to be deplored, that Capt. T. should have repeatedly and constantly withheld information relating to his disorder, that might have been the means of saving a most valuable life. Every hour in the day, he was questioned whether he never had had such complaints before, but he always persevered so strenuously in denying it, that although it was still suspected, yet nothing was known, till knowledge came too late.

Letters

Letters had been dispatched to his family the day after he fell ill; but from the considerable distance, answers unfortunately did not arrive by the return of post, till the day following that of his dissolution. Then, and not before, a letter was received, from which the lines below are extracted.

"About three years ago, when the regiment was in Dublin, he was very ill with violent excruciating pains in his head, attended sometimes with convulsive attacks, for which he was ordered, and took, medicines for the cure of nervous and spasmodic complaints; but they increased to such a degree, that a consultation was held, and the medical gentlemen could not agree as to the cause of his complaint.

"Dr. Boyton, an eminent physician in Dublin, being of opinion that it proceeded from an old venereal taint, recommended a trial of mercury; his advice was followed, and the consequence was, that *as soon as the mercury took effect, the pain in his head, and the epileptic attacks were removed,* and I believe he has ever since, till now, been in uninterrupted health."

*Mill Street, Hanover Square,*
*May* 11, 1810.

( To be Continued. )

